# Clinical onset of atopic eczema: Results from 2 nationally representative British birth cohorts followed through midlife

**DOI:** 10.1016/j.jaci.2019.05.040

**Published:** 2019-09

**Authors:** Katrina Abuabara, Morgan Ye, Charles E. McCulloch, Alice Sullivan, David J. Margolis, David P. Strachan, Lavinia Paternoster, Yik Weng Yew, Hywel C. Williams, Sinéad M. Langan

**Affiliations:** a Program for Clinical Research, Department of Dermatology, University of California, San Francisco School of Medicine, San Francisco, Calif; b Division of Biostatistics, University of California, San Francisco School of Medicine, San Francisco, Calif; c UCL Institute of Education, University College, London, United Kingdom; d Department of Dermatology and Center for Epidemiology and Biostatistics, University of Pennsylvania Perelman School of Medicine, Philadelphia, Pa; e Population Health Research Institute, St George's University of London, London, United Kingdom; f MRC Integrative Epidemiology Unit, Bristol Medical School, University of Bristol, Bristol, United Kingdom; g National Skin Centre, Singapore; h Centre of Evidence Based Dermatology, Faculty of Medicine & Health Sciences, University of Nottingham, Nottingham, United Kingdom; i Faculty of Epidemiology and Population Health, London School of Hygiene and Tropical Medicine, London, United Kingdom

**Keywords:** Atopic eczema, atopic dermatitis, natural history, epidemiology, BCS, British Cohort Study, FLG, Filaggrin, OR, Odds ratio, UK, United Kingdom

## Abstract

**Background:**

Atopic eczema onset is described primarily in early childhood, and the frequency and characteristics of adult-onset disease remain controversial.

**Objective:**

We sought to determine the proportion of subjects who report atopic eczema symptoms between birth and midadulthood and to examine demographic, immunologic, and genetic factors associated with period of symptom onset.

**Methods:**

We conducted a longitudinal study using data from 2 nationally representative community-based birth cohorts from the United Kingdom: the British Cohort Studies 1958 and 1970. Subjects were followed from birth through age 42 to 50 years. The primary outcome was the age period of self-reported atopic eczema symptom onset based on repeated measures of self-reported atopic eczema at each survey wave.

**Results:**

The annual period prevalence of atopic eczema ranged from 5% to 15% in 2 cohorts of more than 17,000 participants each followed from birth through middle age. There was no clear trend in prevalence by age, and among adults reporting active atopic eczema during a given year, only 38% had symptom onset reported in childhood. When compared with subjects whose eczema started in childhood, those with adult-onset disease were more likely to be women, from Scotland or Northern England, of lower childhood socioeconomic group, smokers in adulthood, and less likely to have a history of asthma. In a subanalysis using data from the 1958 cohort only, genetic mutations previously associated with atopic eczema, including filaggrin-null mutations, and allergen-specific IgE were more common among those with childhood-onset disease.

**Conclusion:**

Rates of self-reported atopic eczema remain high after childhood, and adult-onset atopic eczema has different risk factor associations than childhood-onset eczema.

## Graphical abstract

**Figure:**
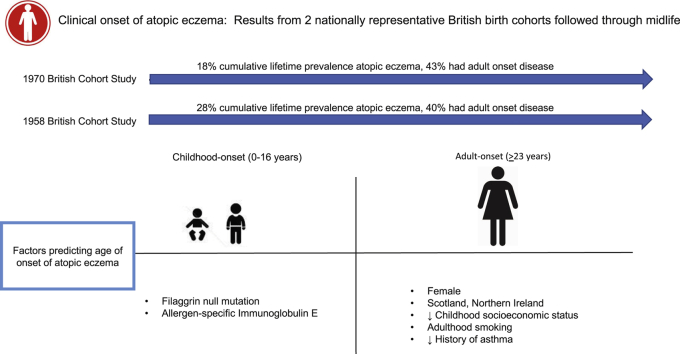

Atopic eczema (also known as atopic dermatitis or just eczema) is the leading cause of skin-related disability,^1^ but most epidemiologic research has focused only on incidence early in life or patterns of disease in childhood.^2^ Recent data suggest that atopic eczema is also common among adults, but whether these trends are due to increasing persistence of disease or new-onset disease later in life is unclear.^3^, ^4^, ^5^ Atopic eczema is known to wax and wane over time, yet there are limited longitudinal data on patterns of disease activity over the life course. Cross-sectional studies have reported proportions of adult-onset atopic eczema ranging from 13% to 60%.^6^, ^7^, ^8^, ^9^, ^10^, ^11^, ^12^, ^13^, ^14^, ^15^ The validity of these estimates have been questioned because of the potential for recall bias (adults might not accurately recall whether they had eczema as children) or the possibility that disease expression in adulthood is due to migration from low- to high-prevalence climates.^16^ In addition, studies of dermatology clinic populations suggest that there might be important genetic and phenotypic differences in patients with adult-onset disease, but these might not be representative of the general population and are controversial for the reasons stated above. Data from population-based longitudinal birth cohorts are needed to understand the patterns and predictors of atopic eczema presentation across the life course.

It is important to understand the epidemiology of adult-onset atopic eczema for a number of reasons. First, because most diagnostic criteria specify that disease begins early in childhood, patients and providers might feel uncertain of the diagnosis in adults with new-onset disease. Although additional testing is often appropriate to rule out differential diagnoses,^17^ if many adults do not meet the diagnostic criteria developed for children, they might be subject to anxiety about the lack of a clear diagnosis, excessive testing, and limited access to new treatment options.^18^

Second, if risk factors for adult-onset atopic eczema differ, this raises the possibility of a different subtype of atopic eczema and could help to elucidate differences in disease pathophysiology and drivers of disease activity.

Finally, understanding whether childhood-onset and adult-onset atopic eczema differ is important for refining preventative and treatment strategies. The latter is particularly timely because many new small molecules and biologic agents are currently under development and clinical testing for use in patients with atopic eczema.^18^

Using 2 large cohorts followed from birth for 4 to 5 decades that are representative of the general United Kingdom (UK) population, we sought to determine the proportion of patients with symptoms of self-reported atopic eczema in childhood and adulthood and examine factors associated with period of onset.

## Methods

**Fig 1:**
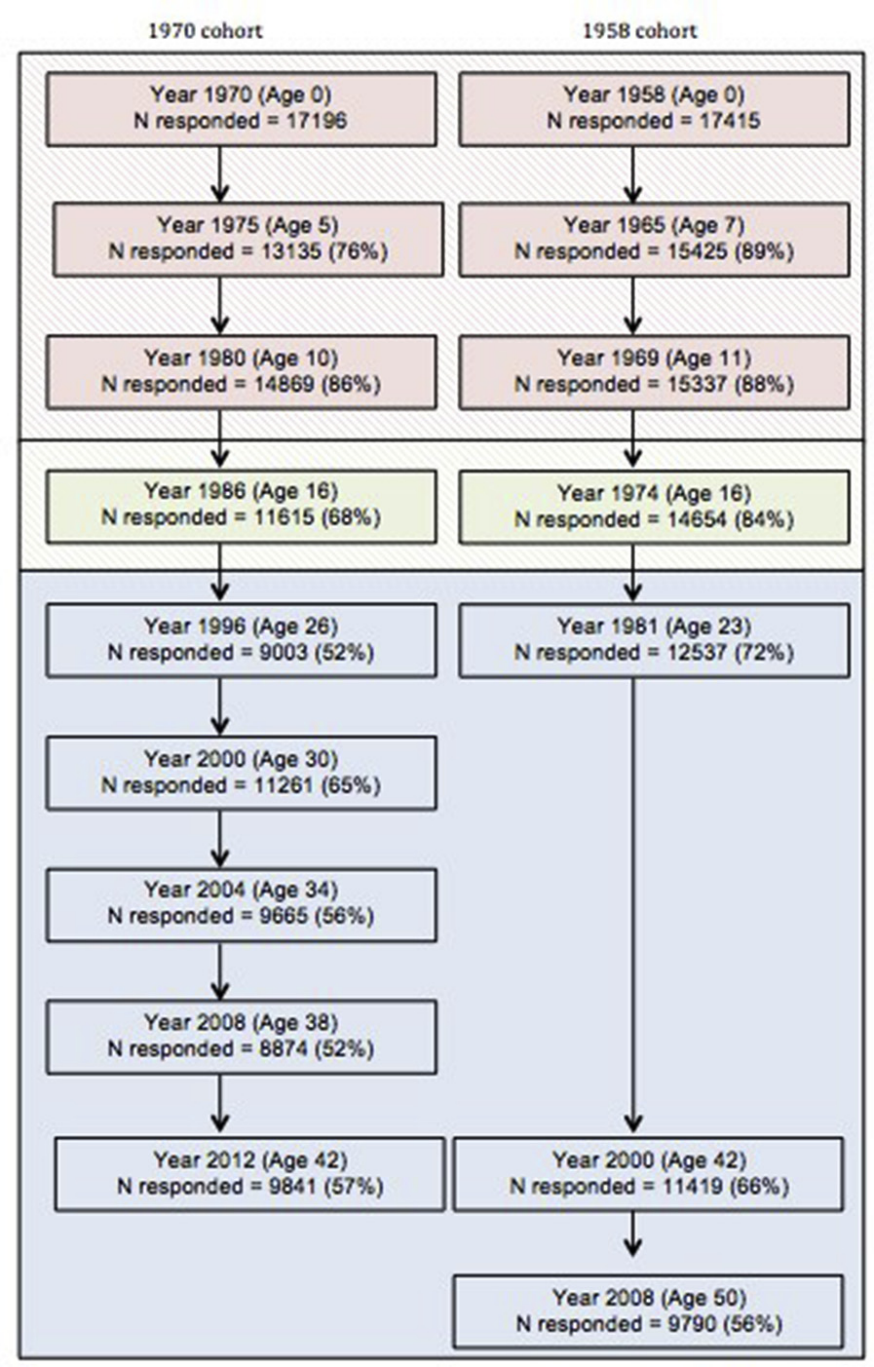
Flow chart of ages at which atopic eczema activity was assessed. Percentages represent the proportion of the original sample assessed at each age. Red shading indicates ages used to define childhood-onset atopic eczema, and blue shading indicates ages used to define adult-onset atopic eczema. Data from age 16 years were included with childhood-onset disease in a sensitivity analysis.

We performed a longitudinal cohort study using data from the 1958 and 1970 British Cohort Studies (BCS1958 and BCS70), which are ongoing multidisciplinary studies that include 17,196 and 17,415 babies born in Great Britain during one week in March 1958 and March 1970, respectively.^19^, ^20^ There have been 8 to 9 subsequent waves of follow-up in each cohort at approximately 5- to 10-year intervals (Fig 1). In the 1958 study, waves at ages 33, 46, and 55 years did not include data on atopic eczema and thus were not included in the analysis. Additional information on response patterns in both cohorts has been reported elsewhere.^21^, ^22^

### Outcomes

The primary outcome was parent-reported or self-reported period prevalence of atopic eczema based on a standardized question asking about “eczema” during or before the past year or since the last survey at each wave of follow-up (see Table E1 in this article's Online Repository at www.jacionline.org). In descriptive analyses this measure coincided well with standardized clinical examinations among children in the 1958 birth cohort,^23^, ^24^ and a similar question has been shown to have high sensitivity and specificity for physician-diagnosed atopic eczema in US children and adults.^25^ We categorized subjects who reported atopic eczema into 2 groups: those whose first report of atopic eczema occurred in childhood (positive parental report during *or* before the last year at age 5-7 and/or 10-11 years) and those with adult-onset atopic eczema (first report of atopic eczema at age ≥23 years). For the primary analysis, we did not include atopic eczema data from age 16 years because it is considered a transitional period between pediatric and adult care in the UK and the 1958 cohort only asked about annual period prevalence (rather than period and lifetime prevalence at that age). In sensitivity analyses data from age 16 years were included.

### Covariates

Additional covariates were chosen based on prior literature showing an association with atopic eczema.^23^, ^26^, ^27^ These included sex, ethnic group, history of any breast-feeding, region of residence in childhood, region of residence in adulthood (at age 42 years), childhood smoke exposure (either parent reporting current smoking during childhood surveys), smoking in adulthood (personal report of current smoking on any of the surveys in adulthood), household size (categorized into ≤3 persons and ≥4 persons), *in utero* smoke exposure (mother reported any smoking during pregnancy), birth weight, and the Registrar General's designation of social class (a standard measure based on the father's highest occupational status reported on any survey at ages 0-10/11 years for childhood and a subject's own occupation at ages 23-50 years for adulthood). Personal history of asthma or allergic rhinitis/hay fever was based on questions repeated at multiple ages (see Table E2 in this article's Online Repository at www.jacionline.org). Data on parental history of asthma and hay fever were only available in the 1970 cohort and were based on either parent's report of either condition at age 5 years.

### Primary analysis

In both cohorts we estimated the cumulative lifetime prevalence and age-specific period prevalence. We also calculated the proportion of subjects with childhood-onset versus adult-onset disease among those who reported active atopic eczema in adulthood. We used multivariable logistic regression to test for differences in demographic and risk factors between (1) childhood-onset and no atopic eczema, (2) adult-onset and no atopic eczema, and (3) childhood-onset and adult-onset atopic eczema. After examining the regression results for consistency in each cohort separately, we conducted a meta-analysis of individual participant data, assuming fixed effects across studies to account for the clustering of participants within cohorts.^28^

### Subgroup analysis and biospecimen data

At the age of 44 to 45 years, 5974 subjects in the 1958 cohort were followed up with a biomedical examination and blood sampling.^29^ For the subgroup of the 1958 cohort who had biospecimen data available, we repeated regressions including variables for the presence of any filaggrin *(FLG)*–null mutation and a non-*FLG* genetic risk score, total IgE level, and allergen-specific IgE level modeled as 3-level categorical variables derived as tertiles. The total concentration of serum IgE antibodies and the presence of specific IgE to house dust mite, mixed grass pollen, and cat fur were ascertained by using the HYTEC enzyme immunoassay (HYCOR Biomedical, Garden Grove, Calif), with a detection threshold of 0.35 kU/L.^30^ Four common null mutations of the *FLG* gene that have been associated with risk of atopic dermatitis in European populations^31^, ^32^ were genotyped directly by LGC Genomics (Berlin, Germany) using KASP genotyping technology. *FLG*-null status was defined as the presence of 1 or more risk variants of rs61816761 (R501X), rs150597413 (S3247X), rs558269137 (2282del4), or rs138726443 (R2447X, formerly rs386430951). An additional 29 variants outside the *FLG* region were selected for inclusion in a polygenic risk score based on previously published associations with atopic dermatitis (please see the Methods section in this article's Online Repository at www.jacionline.org for additional description of the methods used and a full list of references). A non-*FLG* genetic risk score was generated as the sum of imputed allele dosages for the risk-associated variant at each of these single nucleotide polymorphisms.

### Sensitivity analyses

For the primary analysis, we did not include atopic eczema data from age 16 years, as described above. In a preplanned sensitivity analysis we tested the effect of this decision on our results by including subjects who reported atopic eczema during the past year at age 16 years with the childhood-onset group. We also examined the potential for misclassification bias by restricting the sample to those who reported having seen a physician for their eczema in the past year and had no history of self-reported psoriasis or contact dermatitis.

### Missing data

We explored patterns of missing data throughout follow-up. For the primary analysis, we included only subjects with at least 1 survey response in childhood and 1 survey response in adulthood (Fig 1). Additionally, to explore the effect of missing data, we performed multiple imputation in each cohort separately with iterative chained equations to impute missing exposure, outcome, and covariate data. Thirty imputed data sets were generated, and average results from repeated analyses were compared with the complete case analysis. All statistical analyses were conducted with Stata software (version 14; StataCorp, College Station, Tex).

## Results

At birth, 17,196 subjects were recruited into the 1970 cohort, and 17,415 subjects were recruited into the 1958 cohort. There were intermittent missing data and attrition in both cohorts over time; 56% to 57% of the original birth sample responded to the last wave of follow-up (Fig 1). Data on atopic eczema in both childhood and adulthood were available for 11,886 members of the 1970 cohort and 13,143 members of the 1958 cohort; demographic characteristics and missing covariate data are shown in Table I and Table E3 in this article's Online Repository at www.jacionline.org.

**Table I:** Patient characteristics by AE and period of onset

	1970 cohort			1958 cohort		
	No AE (n = 8,611)	Any AE (n = 3,275 [28% of cohort])		No AE (n = 10,825)	Any AE (n = 2,318 [18% of cohort])	
		Childhood onset (n = 1,972 [60% of those with AE])	Adult onset (n = 1,303 [40% of those with AE])		Childhood onset (n = 1,313 [57% of those with AE])	Adult onset (n = 1,005 [43% of those with AE])
	No. (%)			No. (%)		
Sex						
Male	4,406 (51.2)	996 (50.5)	467 (35.8)	5,594 (51.7)	663 (50.5)	383 (38.1)
Female	4,205 (48.8)	976 (49.5)	836 (64.2)	5,231 (48.3)	650 (49.5)	622 (61.9)
Ethnicity						
European, white	7,164 (96.3)	1,709 (96.8)	1,071 (96.5)	8,749 (99.0)	1,147 (98.7)	822 (98.9)
Other	272 (3.7)	56 (3.2)	39 (3.5)	88 (1.0)	15 (1.3)	9 (1.1)
Region of residence in childhood						
Southern England	2,552 (33.4)	725 (40.3)	418 (36.5)	3,193 (29.5)	431 (32.8)	311 (30.9)
Central England	2,112 (27.6)	487 (27.0)	316 (27.6)	3,224 (29.8)	433 (33.0)	303 (30.1)
Northern England	2,988 (39.0)	589 (32.7)	411 (35.9)	4,408 (40.7)	449 (34.2)	391 (38.9)
Region of residence at age 42 y						
Southern England	2,283 (37.0)	636 (43.3)	402 (38.5)	3,136 (37.1)	429 (40.5)	365 (39.9)
Central England	1,671 (27.1)	404 (27.5)	290 (27.8)	2,199 (26.0)	302 (28.5)	261 (28.5)
Northern England	2,215 (35.9)	429 (29.2)	351 (33.7)	3,115 (36.9)	327 (30.9)	289 (31.6)
Social class in childhood						
I/II	3,049 (35.4)	891 (45.2)	489 (37.6)	3,072 (28.5)	455 (34.7)	306 (30.5)
IIIa/b	4,905 (57.0)	974 (49.4)	719 (55.2)	6,597 (61.2)	749 (57.1)	603 (60.2)
IV/V	648 (7.5)	106 (5.4)	94 (7.2)	1,104 (10.2)	107 (8.2)	93 (9.3)
Social class in adulthood						
I/II	4,326 (53.2)	1,156 (61.1)	720 (57.4)	4,573 (43.7)	607 (47.6)	482 (49.6)
IIIa/b	3,003 (36.9)	612 (32.3)	454 (36.2)	4,671 (44.7)	536 (42.1)	402 (41.4)
IV/V	801 (9.9)	125 (6.6)	80 (6.4)	1212 (11.6)	131 (10.3)	87 (9.0)
Household size						
≤3 Persons	821 (10.7)	183 (10.1)	121 (10.6)	857 (8.9)	115 (9.3)	75 (8.4)
≥4 Persons	6,845 (89.3)	1,626 (89.9)	1,022 (89.4)	8,766 (91.1)	1,119 (90.7)	821 (91.6)
Smoking during pregnancy						
No	4,635 (54.1)	1,155 (58.8)	730 (56.2)	7,096 (66.3)	910 (70.3)	669 (67.6)
Any	3,934 (45.9)	809 (41.2)	570 (43.8)	3,600 (33.7)	384 (29.7)	320 (32.4)
Childhood smoke exposure						
No	2,616 (34.2)	691 (38.2)	396 (34.7)	2,173 (27.2)	316 (31.9)	215 (28.4)
Any	5,025 (65.8)	1,117 (61.8)	745 (65.3)	5,805 (72.8)	675 (68.1)	541 (71.6)
Adult smoking						
No	4,647 (54.1)	1,095 (55.6)	649 (49.8)	6,076 (56.2)	764 (58.3)	533 (53.0)
Any	3,950 (45.9)	875 (44.4)	654 (50.2)	4,736 (43.8)	547 (41.7)	472 (47.0)
Atopy						
History of asthma	1,451 (16.9)	627 (31.8)	378 (29.0)	2,362 (21.8)	485 (36.9)	322 (32.0)
History of allergic rhinitis/hay fever	3,160 (36.7)	1,090 (55.3)	684 (52.5)	3,070 (28.4)	648 (49.4)	416 (41.4)
Parental history of atopy	1,563 (22.3)	660 (39.3)	281 (27.1)	—	—	—
Birth weight (kg), mean (SD)	3.3 (0.5)	3.3 (0.5)	3.3 (0.5)	3.3 (0.5)	3.4 (0.5)	3.3 (0.5)
Breast-feeding						
No	4,851 (63.7)	1,020 (56.9)	687 (60.5)	3,193 (32.2)	338 (26.9)	283 (30.9)
Any	2,766 (36.3)	773 (43.1)	449 (39.5)	6,729 (67.8)	920 (73.1)	634 (69.1)
*FLG*-null mutations∗						
No	—	—	—	3,689 (90.3)	446 (79.1)	337 (86.9)
Any	—	—	—	398 (9.7)	118 (20.9)	51 (13.1)
Non-*FLG* SNPs∗						
<25 Risk alleles	—	—	—	1,196 (29.3)	124 (22.0)	97 (25.0)
25-28 Risk alleles	—	—	—	1,536 (37.6)	189 (33.5)	139 (35.8)
>28 Risk alleles	—	—	—	1,355 (33.2)	251 (44.5)	152 (39.2)
Total IgE∗						
<30 kU/L	—	—	—	2,157 (52.8)	239 (42.4)	184 (47.4)
30-99 kU/L	—	—	—	1,154 (28.2)	176 (31.2)	119 (30.7)
≥100 kU/L	—	—	—	776 (19.0)	149 (26.4)	85 (21.9)
Allergen-specific IgE∗						
<0.35 kU/L	—	—	—	3,007 (73.6)	321 (56.9)	260 (67.0)
0.35-3.5 kU/L	—	—	—	369 (9.0)	51 (9.0)	37 (9.5)

*AE*, Atopic eczema; *SNP*, single nucleotide polymorphism.

∗ Data were only available for a subset of the 1958 cohort (n = 5,039).

**Fig 2:**
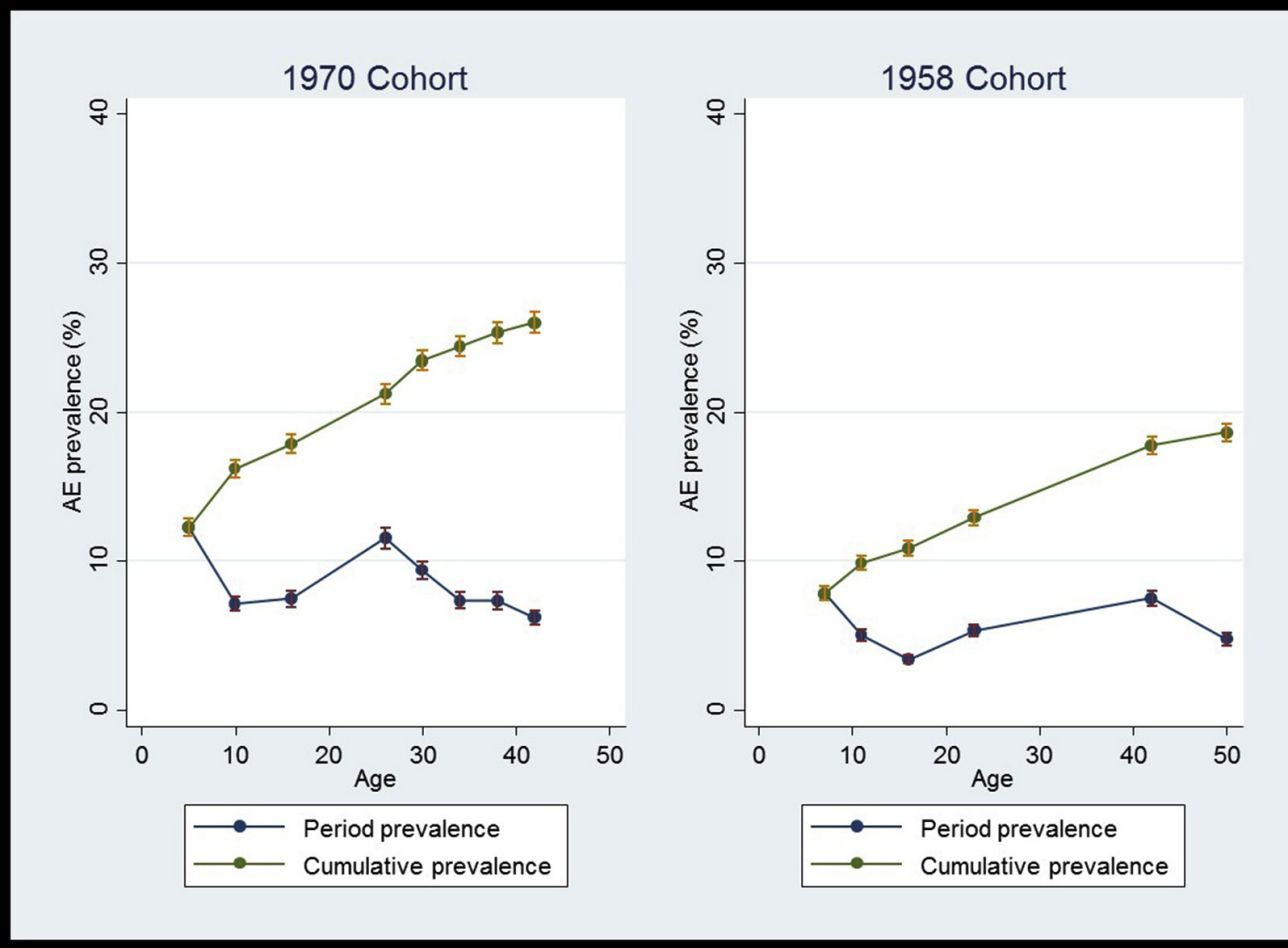
Atopic eczema *(AE)* period prevalence and cumulative lifetime prevalence by age and cohort. *Prevalence from age 0 to 5 years for the 1970 cohort and age 0 to 7 years for the 1958 cohort. *Bars* represent 95% CIs.

**Fig 3:**
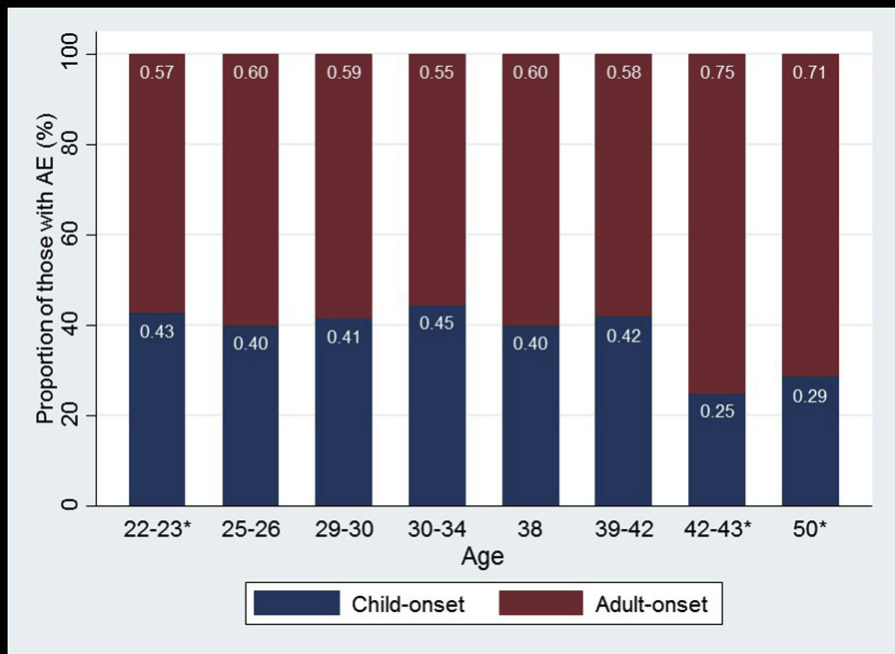
Proportion of subjects with symptom onset in adulthood among those with active atopic eczema *(AE)* at each survey wave in adulthood. Note: Age periods marked by *asterisks* are from the 1958 cohort; the remainder are from the 1970 cohort. Childhood-onset disease was defined as first report at age 0 to 11 years, and adult-onset disease was defined as first report after age 22 to 23 years.

Consistent with international trends, atopic eczema was more common in the 1970 cohort: the cumulative lifetime prevalence of atopic eczema was 28% in the 1970 cohort and 18% in the 1958 cohort. Among those with atopic eczema at any time point, 40% and 43% reported disease for the first time in adulthood in the 1970 and 1958 cohorts, respectively. The period prevalence of atopic eczema ranged from 7% to 14% during any given period in childhood and 5% to 12% during any given period in adulthood (see Table E1), and there was no clear trend across ages in either cohort (Fig 2). Among those who reported atopic eczema activity at each survey wave in adulthood, the majority (mean, 62%) did not have a report of eczema during childhood (Fig 3).

The strength of association from multivariate regression models comparing subjects with childhood-onset atopic eczema and adult-onset atopic eczema with subjects without atopic eczema differed, as is evidenced by the results of the regression model directly comparing those with adult-onset disease with those with childhood-onset disease (Table II). We found that subjects with adult-onset atopic eczema were more likely to be women, to be from Northern geographic areas in the UK, to have been of lower social class in childhood, and to have been smokers during adulthood but were less likely to have a history of asthma (Table II).

**Table II:** Multivariable regression results (complete case analysis)

	OR (95% CI)			*P* value
	Childhood-onset vs no AE (n = 17,373)	Adult-onset vs no AE (n = 12,956)	Adult-onset vs childhood-onset AE (n = 12,956)	
Sex				
Male	Reference	Reference	Reference	
Female	1.04 (0.95-1.13)	**1.75 (1.56-1.98)**	**1.66 (1.44-1.92)**	**<.001**
Ethnicity				
European, white	Reference	Reference	Reference	
Other	0.86 (0.65-1.15)	1.07 (0.73-1.57)	1.23 (0.77-1.97)	.391
Region of early childhood residence				
Southern England	Reference	Reference	Reference	
Central England/Wales	0.91 (0.82-1.02)	0.88 (0.73-1.08)	0.95 (0.75-1.20)	.651
Northern England/Scotland	**0.77 (0.70-0.86)**	1.03 (0.83-1.29)	**1.31 (1.01-1.71)**	**.045**
Region of residence at age 42 y				
Southern England	—	Reference	Reference	
Central England/Wales	—	1.17 (0.96-1.42)	1.14 (0.90-1.43)	.281
Northern England/Scotland	—	0.87 (0.70-1.09)	0.92 (0.71-1.20)	.551
Highest social class in childhood∗				
I/II	Reference	Reference	Reference	
III	**0.80 (0.73-0.88)**	1.00 (0.88-1.14)	**1.23 (1.05-1.43)**	**.009**
IV/V	**0.70 (0.58-0.85)**	0.99 (0.77-1.26)	**1.38 (1.01-1.89)**	**.044**
Highest social class in adulthood∗				
I/II	—	Reference	Reference	
III	—	0.92 (0.81-1.04)	1.00 (0.86-1.17)	.956
IV/V	—	**0.75 (0.58-0.96)**	0.90 (0.66-1.22)	.489
Household size in early childhood				
≤3 Persons	Reference	Reference	Reference	
≥4 Persons	1.01 (0.87-1.17)	1.08 (0.88-1.31)	1.10 (0.87-1.40)	.439
*In utero* smoke exposure				
No	Reference	Reference	Reference	
Any	0.93 (0.84-1.03)	0.94 (0.82-1.07)	0.99 (0.84-1.16)	.915
Childhood smoke exposure				
No	Reference	Reference	Reference	
Any	0.93 (0.85-1.03)	1.02 (0.89-1.16)	1.07 (0.91-1.26)	.400
Adult smoking				
No	—	Reference	Reference	
Any	—	**1.26 (1.13-1.42)**	**1.20 (1.04-1.38)**	**.013**
Asthma				
No	Reference	Reference	Reference	
Any	**1.85 (1.68-2.04)**	**1.45 (1.27-1.66)**	**0.79 (0.68-0.93)**	**.004**
Allergic rhinitis/hay fever				
No	Reference	Reference	Reference	
Any	**1.81 (1.65-1.98)**	**1.59 (1.41-1.80)**	0.90 (0.77-1.04)	.141
Birth weight				
Per kilogram increase	**1.09 (1.00-1.19)**	1.01 (0.90-1.13)	0.90 (0.79-1.04)	.156
Breast-feeding				
No	Reference	Reference	Reference	
Any	**1.18 (1.07-1.29)**	1.11 (0.98-1.26)	0.94 (0.81-1.09)	.411

Boldface indicates *P* < .05.

*AE*, Atopic eczema.

∗ Registrar General's social class: *I*, professional; *II*, managerial and technical; *III*, skilled; *IV*, partly skilled; and *V*, unskilled.

In a subgroup analysis using data from 3365 subjects in the 1958 cohort who were part of the biomedical follow up at age 44 to 45 years and had atopic eczema, genetic, IgE, and covariate data available, we examined rates of known risk alleles for AD and both total IgE and allergen-specific IgE levels. We found that 21% of those with childhood-onset disease, 13% with adult-onset disease, and 10% without any history of atopic eczema had at least 1 *FLG*-null mutation (Table I). Both childhood-onset and adult-onset atopic eczema were associated with *FLG*-null mutations, but the association was stronger for childhood-onset than adult-onset atopic eczema in multivariable analyses (odds ratio [OR], 2.73 [95% CI, 2.06-3.63] and 1.49 [95% CI, 1.01-2.19], respectively; Table III). A high non-*FLG* genetic risk score predicted childhood-onset atopic eczema, but there was little evidence for an association between the non-*FLG* genetic risk score and adult-onset disease (OR, 1.81 [95% CI, 1.37-2.40] and 1.18 [95% CI, 0.85-1.64], respectively; Table III). Similarly, a high allergen-specific IgE levels predicted childhood-onset atopic eczema, but there was little evidence for an association between the allergen-specific IgE and adult-onset disease (OR, 1.90 [95% CI, 1.32-2.74] and 0.86 [95% CI, 0.54-1.36], respectively; Table III).

**Table III:** Multivariable regression results from subanalysis with genetic data from the 1958 birth cohort∗

	OR (95% CI)			*P* value
	Child-onset vs no AE (n = 3444)	Adult-onset vs no AE (n = 3365)	Adult-onset vs child-onset AE (n = 3365)	
*FLG*-null mutations				
No	Reference	Reference	Reference	
Any	**2.73 (2.06-3.63)**	**1.49 (1.01-2.19)**	**0.54 (0.35-0.83)**	**.006**
Non-*FLG* SNPs				
<25 Risk alleles	Reference	Reference	Reference	
25-28 Risk alleles	1.17 (0.87-1.58)	1.07 (0.77-1.48)	0.87 (0.57-1.32)	.507
>28 Risk alleles	**1.81 (1.37-2.40)**	1.18 (0.85-1.64)	**0.64 (0.43-0.97)**	**.036**
Total IgE				
<30 kU/L	Reference	Reference	Reference	
30-99 kU/L	1.14 (0.83-1.56)	1.08 (0.76-1.54)	0.96 (0.61-1.51)	.866
≥100 kU/L	0.93 (0.63-1.38)	1.08 (0.68-1.71)	1.22 (0.68-2.17)	.503
Allergen-specific IgE				
<0.35 kU/L	Reference	Reference	Reference	
0.35-3.5 kU/L	1.09 (0.69-1.71)	1.05 (0.63-1.74)	0.94 (0.49-1.78)	.846
≥3.5 kU/L	**1.90 (1.32-2.74)**	0.86 (0.54-1.36)	**0.44 (0.25-0.77)**	**.004**
Sex				
Male	Reference	Reference	Reference	
Female	0.98 (0.79-1.22)	**1.67 (1.27-2.19)**	**1.71 (1.23-2.39)**	**.002**
Region of early childhood residence				
Southern England	Reference	Reference	Reference	
Central England/Wales	0.84 (0.64-1.10)	1.03 (0.68-1.57)	1.17 (0.71-1.94)	.535
Northern England/Scotland	**0.65 (0.49-0.85)**	1.30 (0.81-2.07)	1.64 (0.92-2.93)	.094
Region of residence at age 42 y				
Southern England	—	Reference	Reference	
Central England/Wales	—	1.15 (0.77-1.70)	1.16 (0.72-1.87)	.550
Northern England/Scotland	—	0.67 (0.42-1.08)	0.85 (0.47-1.53)	.585
Social class in childhood†				
I/II	Reference	Reference	Reference	
IIIa/b	0.99 (0.78-1.25)	1.05 (0.78-1.41)	1.08 (0.76-1.55)	.662
IV/V	0.79 (0.50-1.26)	1.29 (0.79-2.09)	1.61 (0.85-3.06)	.142
Highest social class in adulthood†				
I/II	—	Reference	Reference	
III	—	0.85 (0.64-1.13)	0.77 (0.54-1.08)	.131
IV/V	—	0.74 (0.44-1.22)	0.75 (0.40-1.41)	.369
Household size in early childhood				
≤3 Persons	Reference	Reference	Reference	
≥4 Persons	0.87 (0.59-1.27)	1.14 (0.69-1.89)	1.30 (0.71-2.38)	.388
*In utero* smoke exposure				
No	Reference	Reference	Reference	
Any	0.92 (0.71-1.19)	0.99 (0.73-1.35)	1.08 (0.74-1.58)	.697
Childhood smoke exposure				
No	Reference	Reference	Reference	
Any	0.93 (0.73-1.19)	0.93 (0.69-1.26)	0.98 (0.68-1.42)	.910
Adult smoking				
No	—	Reference	Reference	
Any	—	1.01 (0.77-1.33)	0.94 (0.68-1.32)	.739
Asthma				
No	Reference	Reference	Reference	
Any	**1.53 (1.21-1.94)**	**1.38 (1.03-1.85)**	0.88 (0.62-1.25)	.482
Allergic rhinitis/hay fever				
No	Reference	Reference	Reference	
Any	**1.62 (1.26-2.07)**	**1.54 (1.14-2.06)**	0.95 (0.66-1.36)	.761
Birth weight				
Per kilogram increase	0.98 (0.79-1.21)	1.03 (0.80-1.34)	1.03 (0.75-1.41)	.870
Breast-feeding				
No	Reference	Reference	Reference	
Any	1.18 (0.91-1.51)	0.97 (0.73-1.30)	0.81 (0.57-1.17)	.264

Boldface indicates *P* < .05

*AE*, Atopic eczema; *SNP*, single nucleotide polymorphism.

∗ Does not include data from survey at age 50 years.

† Registrar General's social class: *I*, professional; *II*, managerial and technical; *III*, skilled; *IV*, partly skilled; and *V*, unskilled.

Analyses after multiple imputation to address missing data showed similar results (see Table E4 in this article's Online Repository at www.jacionline.org). In a sensitivity analysis using data from age 16 years, we found that an additional 260 subjects would be classified as having childhood-onset disease in the 1970 cohort as would an additional 193 children in the 1958 cohort. The overall proportion with childhood-onset disease remained near 60% in both cohorts, and results of regression analyses did not substantiatively change (see Table E5 in this article's Online Repository at www.jacionline.org). Finally, when we excluded patients with a history of contact dermatitis or psoriasis, and respondents who did not report seeing a physician in the past year for their atopic eczema (see Table E6 in this article's Online Repository at www.jacionline.org), we again found similar results (see Table E7 in this article's Online Repository at www.jacionline.org).

## Discussion

Using 2 large population-based cohorts followed from birth into midlife, we found the period prevalence of self-reported atopic eczema was 5% to 14%. One of the defining characteristics of childhood atopic eczema is early age at onset; however, the majority of those reporting symptoms in adulthood did not have disease onset in childhood. When comparing those with childhood-onset and adult-onset atopic eczema, we found differences in demographic characteristics, atopic comorbidities, IgE profiles in adulthood, and genetic risk factors. Our findings help to address the gap in knowledge about the epidemiology of adult atopic eczema and suggest that there might be different subtypes of adult disease that warrant additional characterization.

### Strengths and limitations

**Fig E1:**
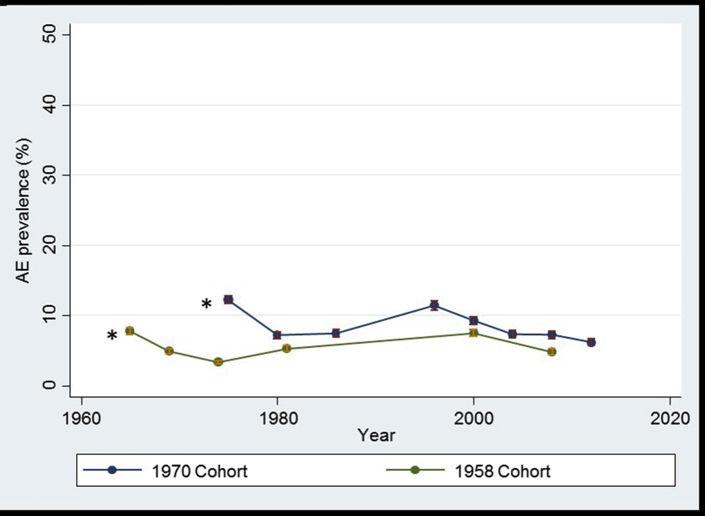


Our study is unique in that there is prospective follow-up of subjects residing in the UK from birth through middle age. The data come from 2 large community-based cohorts broadly representative of the UK general population. Consistent with previous reports and international trends,^33^, ^34^, ^35^ we found that the overall prevalence of atopic eczema increased between 1958 and 1970, but there did not appear to be a difference in trends across calendar years (see Fig E1 in this article's Online Repository at www.jacionline.org). Two population-based mail surveys in the United States and Italy also found high rates of adult-onset disease (54% and 60% of the population respectively)^6^, ^7^ but have been questioned because of the possibility for poor recall of childhood disease or migration to new climates.^16^ These biases are unlikely to affect our estimates because subjects in our cohorts were born in the UK and followed with repeated assessments from birth through midlife.

We likely found a lower proportion of subjects with early-onset disease because our data included a longer duration of prospective follow-up than prior studies.^36^ For example, an older study using data available through age 23 years from the 1958 BCS concluded that of the 870 cases by the age of 16 years, 66% had age of onset by the age of 7 years.^37^ By comparison, using the same initial data, now with extended follow-up through age 50 years, we found only 41% had onset of symptoms by age 7 years. Longitudinal studies of asthma have similarly found higher rates of late-onset and recurrent disease with longer periods of follow-up.^38^, ^39^

A limitation of our study is that our outcome of atopic eczema was based on parental report or self-report, and it is likely that some patients were misclassified. Misclassification could include other forms of eczema, including stasis dermatitis and irritant contact dermatitis in adults. Nearly all of the population-based epidemiologic literature on atopic eczema has relied on self-reported assessment of disease, and prior studies have shown that self-report performs reasonably well: in a multicenter US study with a physician's diagnosis as the gold standard, the positive predictive value of self-report was 0.87 (95% CI, 0.78-0.96) in children and 0.76 (95% CI, 0.64-0.85) in adults.^25^ Of note, it performed better for children than adults, and the study was conducted with dermatology clinic patients in whom the prevalence of atopic eczema was greater than in the general population, meaning the estimates could be slightly inflated. Additional analyses to examine the potential for misclassification, including restricting our sample to those who reported having seen a physician for their atopic eczema and never reported contact dermatitis and psoriasis, were similar to primary regression results (see Table E7). Although these results do not rule out the potential for misclassification bias, they suggest that the magnitude of bias is likely to be small. Furthermore, as described in more detail below, our findings on *FLG* mutations, IgE levels, and demographic factors are similar to smaller studies of clinical populations with physician-diagnosed atopic eczema.^15^, ^16^, ^40^, ^41^

An additional limitation of our study is that surveys were fielded at multiyear intervals, and we cannot rule out the possibility that atopic eczema might be underreported. For example, some parents might not recall a history of early or mild atopic eczema when asked at age 5 to 7 years of their child's life; however, the recall is likely to be superior to that on surveys of adults asked about their own early childhood disease decades later.^42^ Similarly, many of the adult surveys only asked about atopic eczema during the past year (as shown in Table E1), and therefore our results might underestimate adult-onset atopic eczema. Detailed phenotypic assessments of participants to detect atopic eczema at frequent intervals would be desirable, but they are impractical in large population-based cohorts followed for more than 40 years.

Finally, as with any long-term study, the data are limited by attrition over time. Prior research has shown that in the 1970 cohort there is a weak predictive effect of sex and socioeconomic status on response: men from lower social backgrounds with less educated parents are less likely to respond, which has previously been described in detail.^43^ Because the cohort was not explicitly designed to study atopic disease, it is unlikely that attrition was differential by atopic eczema status. Nonetheless, to address missing data issues, we performed multiple imputation and found results that were consistent with those of the complete case analysis.

### Implications for research and clinical practice

Our results highlight the need for additional research to better characterize adult eczema and understand whether pathophysiology differs by age of onset. Atopic eczema is known to have a multifactorial cause, and we found genetic, immunologic, demographic, and risk factor differences between those with childhood-onset and those with adult-onset disease. Only a few other smaller studies have explicitly addressed age-associated differences in patients with atopic eczema, and their findings are largely consistent with our results. Studies from dermatology clinic populations in Germany and the United States also found that those with self-reported adult-onset disease were more likely to be female^41^ and less likely to have a personal or family history of atopic disease,^40^, ^41^ increased IgE levels,^40^, ^44^ or *FLG* mutations^45^, ^46^ but did not find differences by smoking or socioeconomic status.^41^ In contrast, a small case-control study from Taiwan found both current and ever smoking were strong independent risk factors for adult-onset disease,^47^ and a recent meta-analysis found high rates of smoking in adults with AD overall but did not differentiate by age of onset.^48^

Atopic eczema is considered a clinical diagnosis, and the most widely used diagnostic criteria (the Hanifin and Rajka criteria, the UK Working Party criteria refinement of the Hanifin and Rajka criteria, and the American Academy of Dermatology criteria) all include early age at onset and history of atopy.^49^ Clinicians evaluating adults with a potential diagnosis of atopic eczema should recognize that the majority of patients might not have symptom onset in childhood. Moreover, although subjects with adult-onset disease have a greater probability of having a history of other atopic disease than subjects without atopic eczema, asthma was only present in about one third and allergic rhinitis in about one half of patients with atopic eczema in our study (Table I). Diagnostic criteria were developed based on expert opinion among dermatologists whose clinical experience might not reflect the distribution of disease in the general population, and none have been validated in a population-based study of adults.^50^ Our data highlight the need to better understand what is adult “atopic” eczema and to refine diagnostic criteria for use in the general adult population. In the meantime, clinical trials of adult atopic eczema should describe the method by which physicians made the diagnosis (if any) and whether validated diagnostic criteria were used to permit exploration of study heterogeneity and enable subgroup analysis in future meta-analyses.

### Terminology

We choose to use the term *atopic eczema* based on a call for consistency in the literature.^51^ There are regional variations in terminology; in the United Kingdom the term *eczema* is considered more precise than *dermatitis*, whereas in the United States the term *atopic dermatitis* is usually preferred.^51^ In either case use of the term *atopic* has been debated because, even among children, not all disease is associated with increased IgE levels or comorbid atopic conditions, including asthma or rhinitis. Indeed, previous research has suggested that the majority of what is called atopic eczema is not atopic at a population level.^52^ Our findings that adult-onset disease was associated with lower rates of IgE and asthma further call into question the use of the term *atopic* in adult disease; nonetheless, we have continued to use this terminology for consistency and clarity. Future studies might uncover subtypes of adult-onset disease that require new terminology.

### Conclusion

We found that self-reported adult-onset atopic eczema is common among 2 community-based British cohorts. Differences in genetic, demographic, and immunologic profiles between those with childhood-onset and those with adult-onset disease suggest there might be different subtypes of atopic eczema and emphasize the need for better characterization of adult-onset disease and validation of diagnostic tools in this population. These data are particularly timely because dozens of new treatments are under development and clinical testing for AD,^18^ and trial populations selected on the basis of early-onset disease are unlikely to be representative of the general population of adults.

**Clinical implications**

Adult-onset eczema is common and might be less likely to present with other atopic disease.

## Supplementary data

Online Repository text
