# Supplementary material for: Clinical onset of atopic eczema: Results from 2 nationally representative British birth cohorts followed through midlife
Source: J Allergy Clin Immunol. 2019 Sep;144(3):710–9. doi: 10.1016/j.jaci.2019.05.040 (PMC6721832; doi:10.1016/j.jaci.2019.05.040)
Supplement: Online Repository text [file mmc1.docx]

**Supplemental tables and figures**

**Supplemental Table 1**. Summary of atopic eczema variables

| Age | Format | | Question | | Number who answered question | Number who responded positively | |
| --- | --- | --- | --- | --- | --- | --- | --- |
| **1970 Cohort** | | | | | | | |
| Age 5 | Parent interview | | Eczema during or prior to past year? | | 12,183 | 1,495 | 12% |
| Age 10 | Interview & exam | | Eczema during or prior to past year?  Eczema during past year? | | 12,389  12,373 | 1,795  887 | 14%  7% |
| Age 16* | Interview & exam | | Eczema during past year? | | 8,900 | 668 | 8% |
| Age 26 | Postal questionnaire | | Eczema during past year? | | 8,333 | 963 | 12% |
| Age 30 | Computer interview | | Eczema during past year? | | 10,394 | 978 | 9% |
| Age 34 | Computer interview | | Eczema since last survey? | | 8,954 | 662 | 7% |
| Age 38 | Telephone interview | | Eczema currently? | | 8,204 | 604 | 7% |
| Age 42 | Computer interview | | Eczema since last survey? | | 9,093 | 566 | 6% |
| **1958 Cohort** | | | | | | | |
| Age 7 | Interview and exam | Eczema during or prior to past year? | | 14,030 | | 1,100 | 8% |
| Age 11 | Interview and exam | Eczema during or prior to past year?  Eczema during past year? | | 12,991  12,983 | | 917  652 | 7%  5% |
| Age 16* | Interview and exam | Eczema in the past year? | | 13,701 | | 470 | 3% |
| Age 23 | Interview | Eczema in the past year? | | 11,838 | | 634 | 5% |
| Age 42 | Computer Interview | Eczema in the past year? | | 10,788 | | 812 | 8% |
| Age 50 | Postal questionnaire | Eczema currently? | | 9,250 | | 442 | 5% |

Notes: *Data used in sensitivity analysis **Supplemental Table 2**. Summary of asthma and hay fever variables

| Age | Format | | Question | | Number who answered question | Number who responded positively | |
| --- | --- | --- | --- | --- | --- | --- | --- |
| **Asthma - 1970 Cohort** | | | | | | | |
| Age 10 | Exam | | Asthma during or prior to last year? | | 11,984 | 605 | 5% |
| Age 16 | Interview & exam | | Asthma during or prior to last year? | | 5,251 | 649 | 12% |
| Age 26 | Postal questionnaire | | Asthma during or prior to last year? | | 8,333 | 1,022 | 12% |
| Age 30 | Computer interview | | Ever had asthma? | | 10,395 | 1,424 | 14% |
| Age 34 | Computer interview | | Asthma during or prior to last year? | | 8,954 | 936 | 10% |
| Age 38 | Telephone interview | | Asthma currently? | | 8,204 | 853 | 10% |
| Age 42 | Computer interview | | Asthma since last survey? | | 9,091 | 798 | 9% |
| **Asthma - 1958 Cohort** | | | | | | | |
| Age 7 | | Interview and exam | | Ever had asthma? | 14,036 | 431 | 3% |
| Age 11 | | Parent interview | | Ever had asthma or wheezy bronchitis? | 12,863 | 1,596 | 12% |
| Age 16 | | Parent interview | | Ever had asthma or wheezy bronchitis? | 10,744 | 1,257 | 12% |
| Age 23 | | Interview | | Asthma, bronchitis since age 16? | 11,877 | 514 | 4% |
| Age 33 | | Interview | | Ever been told has asthma? | 10,794 | 972 | 9% |
| Age 42 | | Computer interview | | Ever had asthma? | 10,799 | 1,199 | 11% |
| Age 50 | | Questionnaire | | Asthma or wheezy bronchitis currently? | 9,250 | 903 | 10% |
| **Rhinitis/hay fever - 1970 Cohort** | | | | | | | |
| Age 5 | Parent interview | | Hay fever during or prior to last year? | | 12,149 | 536 | 4% |
| Age 10 | Interview & Exam | | Hay fever during or prior to last year? | | 12,338 | 1,663 | 13% |
| Age 16 | Interview & exam | | Hay fever during or prior to last year? | | 8,928 | 2,285 | 26% |
| Age 26 | Postal questionnaire | | Hay fever during or prior to last year? | | 8,333 | 2,478 | 30% |
| Age 30 | Computer interview | | Ever had hay fever? | | 10,395 | 2,694 | 26% |
| Age 34 | Computer interview | | Hay fever since last survey? | | 8,954 | 1,984 | 22% |
| Age 38 | Telephone interview | | Hay fever currently? | | 8,204 | 1,640 | 20% |
| Age 42 | Computer interview | | Hay fever since last survey? | | 9,091 | 1,965 | 22% |
| **Rhinitis/ Hay fever - 1958 Cohort** | | | | | | | |
| Age 7 | | Parent interview | | Hay fever or sneezing attacks ever? | 14,020 | 774 | 6% |
| Age 11 | | Parent interview | | Hay fever during the last year? | 12,934 | 1,048 | 8% |
| Age 16 | | Parent interview | | Hay fever during the last year? | 10,553 | 1,279 | 12% |
| Age 23 | | Interview | | Hay fever during the last year? | 11,825 | 1,960 | 17% |
| Age 33 | | Interview | | Ever suffered from hay fever | 10,772 | 2,204 | 20% |
| Age 42 | | Computer interview | | Ever had hay fever? | 10,799 | 2,223 | 21% |
| Age 50 | | Questionnaire | | Hay fever currently? | 9,250 | 1,255 | 14% |

**Supplemental Table 3**: Cohort characteristics and missing data

|  | 1970 Cohort  N (%) | 1958 Cohort  N (%) |
| --- | --- | --- |
| Overall N | 17196 | 17415 |
|  |  |  |
| Sex |  |  |
| Male | 8908 (51.8) | 9001 (51.7) |
| Female | 8280 (48.2) | 8411 (48.3) |
| Missing | 8 (0.01) | 3 (0.02) |
| Ethnicity |  |  |
| European, Caucasian | 11809 (68.7) | 12019 (69.0) |
| African | 154 (0.9) | 98 (0.6) |
| Indian-Pakastani | 178 (1.0) | 32 (0.2) |
| Other | 179 (1.0) | 30 (0.2) |
| Missing | 4876 (28.4) | 5236 (30.1) |
| Region of residence in childhood |  |  |
| Southern England | 4430 (25.8) | 5365 (30.8) |
| Central England | 3465 (20.2) | 5103 (29.3) |
| Northern England | 4812 (28.0) | 6947 (39.9) |
| Missing | 4489 (26.1) | 0 (0.0) |
| Region of residence at age 42 |  |  |
| Southern England | 3532 (20.5) | 4093 (23.5) |
| Central England | 2460 (14.3) | 2859 (16.4) |
| Northern England | 3122 (18.2) | 3878 (22.3) |
| Missing | 8082 (47.0) | 6585 (37.8) |
| Breastfeeding |  |  |
| No | 7975 (46.4) | 4435 (25.5) |
| Any | 4663 (27.1) | 9587 (55.1) |
| Missing | 4558 (26.5) | 3393 (19.5) |
| Childhood smoke exposure^a^ |  |  |
| No | 4347 (25.3) | 2993 (17.2) |
| Any | 8351 (48.6) | 7953 (45.7) |
| Missing | 4498 (26.2) | 6469 (37.1) |
| Adulthood smoking |  |  |
| No | 6746 (39.2) | 7680 (44.1) |
| Any | 5784 (33.6) | 6004 (34.5) |
| Missing | 4666 (27.1) | 3731 (21.4) |
| Smoking during pregnancy |  |  |
| No | 9210 (53.6) | 11407 (65.5) |
| Any | 7899 (45.9) | 5783 (33.2) |
| Missing | 87 (0.5) | 225 (1.3) |
| Household size^b^ |  |  |
| <=3 persons | 1340 (7.8) | 1213 (7.0) |
| 4+ persons | 11395 (66.3) | 12357 (71.0) |
| Missing | 4461 (25.9) | 3845 (22.1) |
| Atopic history |  |  |
| Personal history of asthma | 2726 (15.9) | 3553 (20.4) |
| Missing | 2911 (16.9) | 1527 (8.8) |
| Personal history of allergic rhinitis/hay fever | 5556 (32.3) | 4511 (25.9) |
| Missing | 2241 (13.0) | 1533 (8.8) |
| Parental history of asthma or allergic rhinitis/hay fever | 2961 (17.2) | N/A |
| Missing | 5584 (32.5) | N/A |
| Social class in childhood |  |  |
| I/II | 5756 (33.5) | 4737 (27.2) |
| IIIa/b | 9592 (55.8) | 10459 (60.1) |
| IV/V | 1750 (10.2) | 1990 (11.4) |
| Missing | 98 (0.6) | 229 (1.3) |
| Social class in adulthood |  |  |
| I/II | 6517 (37.9) | 5864 (33.7) |
| IIIa/b | 4300 (25.0) | 5834 (33.5) |
| IV/V | 1068 (6.2) | 1520 (8.7) |
| Missing | 5311 (30.9) | 4197 (24.1) |
| Birth weight (kg), mean (SD) | 3.3 (0.6) | 3.3 (0.6) |
| Missing | 35 (0.2) | 634 (3.6) |

^a^ At age 5 in the 1970 cohort and age 16 in the 1958 cohort

^b^ At age 5 in the 1970 cohort and age 7 in the 1958 cohort

**Supplemental Table 4.** Multivariable regression results for each cohort individually and imputation results

|  | 1970 | | | | 1958 | | | |
| --- | --- | --- | --- | --- | --- | --- | --- | --- |
|  | Complete case | | | Imputation | Complete case | | | Imputation |
|  | Child-onset vs no AE | Adult-onset vs no AE | Adult-onset vs child-onset AE | Adult-onset vs child-onset AE | Child-onset vs no AE | Adult-onset vs no AE | Adult-onset vs child-onset AE | Adult-onset vs child-onset AE |
|  | N=9310 | N=6600 | N=6600 | N=17196 | N=7245 | N=5800 | N=5800 | N=17415 |
|  | Odds Ratio (95% CI) | | | | Odds Ratio (95% CI) | | | |
| Sex | | | | | | | | |
| Male | Reference | Reference | Reference | Reference | Reference | Reference | Reference | Reference |
| Female | 1.04  (0.93, 1.16) | **1.84**  **(1.56, 2.15)** | **1.79**  **(1.48, 2.16)** | **1.47**  **(1.27, 1.70)** | 1.04  (0.89, 1.20) | **1.67**  **(1.38, 2.02)** | **1.54**  **(1.21, 1.96)** | **1.52**  **(1.30, 1.79)** |
| Ethnicity | | | | | | | | |
| European, Caucasian | Reference | Reference | Reference | Reference | Reference | Reference | Reference | Reference |
| Other | 0.92  (0.67, 1.26) | 0.96  (0.62, 1.50) | 1.00  (0.58, 1.70) | 1.17  (0.81, 1.69) | 1.01  (0.42, 2.43) | 1.44  (0.49, 4.22) | 1.07  (0.28, 4.04) | 1.04  (0.49, 2.22) |
| Region of early childhood residence | | | | | | | | |
| Southern England | Reference | Reference | Reference | Reference | Reference | Reference | Reference | Reference |
| Central England | **0.87**  **(0.75, 1.00)** | 0.86  (0.65, 1.14) | 0.95  (0.69, 1.32) | 1.01  (0.79, 1.29) | 1.02  (0.85, 1.23) | 0.94  (0.70, 1.27) | 0.93  (0.65, 1.34) | 1.01  (0.80, 1.26) |
| Northern England | **0.79**  **(0.69, 0.90)** | 0.91  (0.67, 1.24) | 1.07  (0.74, 1.54) | 1.04  (0.77, 1.41) | **0.79**  **(0.65, 0.95)** | 1.27  (0.91, 1.76) | **1.74**  **(1.14, 2.66)** | 1.22  (0.96, 1.55) |
| Region of early adulthood residence | | | | | | | | |
| Southern England | ----- | Reference | Reference | Reference | ----- | Reference | Reference | Reference |
| Central England | ----- | 1.19  (0.90, 1.57) | 1.13  (0.82, 1.56) | 1.12  (0.86, 1.45) | ----- | 1.16  (0.87, 1.54) | 1.15  (0.80, 1.64) | 1.00  (0.79, 1.26) |
| Northern England | ----- | 1.05  (0.77, 1.44) | 1.14  (0.79, 1.65) | 1.17  (0.86, 1.60) | ----- | **0.66**  **(0.48, 0.92)** | 0.67  (0.44, 1.02) | 0.87  (0.67, 1.13) |
| Social class in childhood | | | | | | | | |
| I/II | Reference | Reference | Reference | Reference | Reference | Reference | Reference | Reference |
| IIIa/b | **0.81**  **(0.72, 0.91)** | 0.99  (0.84, 1.17) | **1.23**  **(1.01, 1.50)** | **1.20**  **(1.04, 1.38)** | 0.89  (0.75, 1.04) | 1.02  (0.83, 1.25) | 1.10  (0.85, 1.42) | 1.18  (0.99, 1.40) |
| IV/V | 0.78  (0.60, 1.02) | 1.00  (0.71, 1.43) | 1.35  (0.87, 2.09) | **1.35**  **(1.00, 1.81)** | 0.74  (0.54, 1.01) | 0.99  (0.68, 1.43) | 1.19  (0.74, 1.93) | **1.36**  **(1.00, 1.87)** |
| Social class in adulthood | | | | | | | | |
| I/II | ----- | Reference | Reference | Reference | ----- | Reference | Reference | Reference |
| IIIa/b | ----- | 1.00  (0.85, 1.19) | 1.06  (0.86, 1.29) | **1.16**  **(1.00, 1.36)** | ----- | 0.86  (0.71, 1.05) | 0.97  (0.76, 1.25) | 0.95  (0.80, 1.13) |
| IV/V | ----- | **0.67**  **(0.47, 0.96)** | 0.90  (0.58, 1.38) | 1.07  (0.79, 1.45) | ----- | 0.86  (0.59, 1.27) | 0.88  (0.54, 1.44) | 0.87  (0.65, 1.17) |
| Household size in early childhood | | | | | | | | |
| <=3 persons | Reference | Reference | Reference | Reference | Reference | Reference | Reference | Reference |
| 4+ persons | 1.06  (0.88, 1.27) | 1.03  (0.80, 1.31) | 0.97  (0.72, 1.30) | 1.02  (0.81, 1.28) | 0.82  (0.63, 1.06) | 1.16  (0.81, 1.66) | 1.50  (0.98, 2.30) | 1.21  (0.92, 1.58) |
| In utero smoke exposure | | | | | | | | |
| No | Reference | Reference | Reference | Reference | Reference | Reference | Reference | Reference |
| Any | 0.99  (0.87, 1.12) | 1.01  (0.85, 1.20) | 0.99  (0.80, 1.21) | 0.97  (0.83, 1.13) | 0.90  (0.75, 1.07) | 0.82  (0.66, 1.02) | 0.90  (0.68, 1.19) | 1.02  (0.84, 1.24) |
| Childhood smoke exposure | | | | | | | | |
| No | Reference | Reference | Reference | Reference | Reference | Reference | Reference | Reference |
| Any | 0.96  (0.84, 1.09) | 1.02  (0.85, 1.21) | 1.01  (0.82, 1.25) | 1.07  (0.92, 1.25) | 0.92  (0.77, 1.09) | 1.00  (0.81, 1.24) | 1.11  (0.85, 1.45) | 1.11  (0.87, 1.43) |
| Adulthood smoking | | | | | | | | |
| No | ----- | Reference | Reference | Reference | ----- | Reference | Reference | Reference |
| Any | ----- | **1.35**  **(1.16, 1.58)** | **1.32**  **(1.10, 1.59)** | **1.22**  **(1.06, 1.41)** | ----- | 1.17  (0.97, 1.42) | 1.09  (0.86, 1.39) | **1.20**  **(1.00, 1.43)** |
| Other atopic history | | | | | | | | |
| History of asthma | **1.90**  **(1.66, 2.16)** | **1.58**  **(1.32, 1.89)** | 0.85  (0.69, 1.05) | **0.81**  **(0.69, 0.96)** | **1.76**  **(1.50, 2.07)** | **1.30**  **(1.05, 1.60)** | **0.73**  **(0.57, 0.94)** | **0.79**  **(0.66, 0.95)** |
| History of allergic rhinitis/hay fever | **1.55**  **(1.38, 1.75)** | **1.62**  **(1.38, 1.90)** | 1.06  (0.87, 1.29) | 0.88  (0.76, 1.02) | **2.02**  **(1.73, 2.35)** | **1.51**  **(1.25, 1.84)** | **0.78**  **(0.61, 1.00)** | **0.74**  **(0.62, 0.87)** |
| Parental history of atopy | **1.90**  **(1.69, 2.15)** | 1.07  (0.89, 1.27) | **0.56**  **(0.46, 0.69)** | **0.57**  **(0.49, 0.66)** | **-----** | **-----** | **-----** | **-----** |
| Birth weight | | | | | | | | |
| Per kg increase | 1.09  (0.98, 1.21) | 0.93  (0.80, 1.08) | **0.82**  **(0.69, 0.99)** | **0.86**  **(0.76, 0.98)** | 1.06  (0.91, 1.23) | 1.13  (0.94, 1.36) | 1.08  (0.86, 1.37) | 0.93  (0.78, 1.10) |
| Breastfeeding | | | | | | | | |
| No | Reference | Reference | Reference | Reference | Reference | Reference | Reference | Reference |
| Any | **1.19**  **(1.06, 1.33)** | **1.18**  **(1.01, 1.39)** | 0.95  (0.79, 1.15) | 0.88  (0.76, 1.02) | 1.15  (0.97, 1.37) | 1.02  (0.83, 1.26) | 0.93  (0.71, 1.21) | 0.84  (0.69, 1.02) |

**Supplemental Table 5:** Sensitivity analysis: Multivariable regression results showing the odds of adult-onset vs childhood-onset AE including data from age 16 in childhood-onset group.

|  | 1970 | | | 1958 | | |
| --- | --- | --- | --- | --- | --- | --- |
|  | Child-onset AE N (%) | Adult-onset AE N (%) | Adult-onset vs child-onset AE OR (95% CI) | Child-onset AE N (%) | Adult-onset AE N (%) | Adult-onset vs child-onset AE OR (95% CI) |
|  | N=2232  (64% of those with AE) | N=1246  (36% of those with AE) | N=6617 | N=1506  (60% of those with AE) | N=997  (40% of those with AE) | N=5800 |
| Sex |  |  |  |  |  |  |
| Male | 1079 (48.3) | 459 (36.8) | Reference | 729 (48.4) | 386 (38.7) | Reference |
| Female | 1153 (51.7) | 787 (63.2) | **1.53 (1.26, 1.84)** | 777 (51.6) | 611 (61.3) | **1.40 (1.10, 1.77)** |
| Ethnicity |  |  |  |  |  |  |
| European, Caucasian | 1911 (96.8) | 1003 (96.4) | Reference | 1310 (98.8) | 783 (98.9) | Reference |
| Other | 63 (3.2) | 37 (3.6) | 1.01 (0.59, 1.73) | 16 (1.2) | 9 (1.1) | 1.36 (0.36, 5.16) |
| Region of residence in childhood |  |  |  |  |  |  |
| Southern England | 800 (39.7) | 389 (36.3) | Reference | 485 (32.2) | 308 (30.9) | Reference |
| Central England | 559 (27.8) | 293 (27.3) | 0.98 (0.70, 1.36) | 493 (32.7) | 295 (29.6) | 0.95 (0.66, 1.36) |
| Northern England | 655 (32.5) | 390 (36.4) | 1.21 (0.84, 1.74) | 528 (35.1) | 394 (39.5) | **1.70 (1.12, 2.57)** |
| Region of residence in adulthood |  |  |  |  |  |  |
| Southern England | 711 (42.5) | 382 (38.4) | Reference | 477 (39.3) | 363 (40.0) | Reference |
| Central England | 473 (28.3) | 272 (27.3) | 1.06 (0.76, 1.46) | 346 (28.5) | 257 (28.3) | 1.09 (0.77, 1.55) |
| Northern England | 489 (29.2) | 341 (34.3) | 1.04 (0.72, 1.51) | 392 (32.3) | 288 (31.7) | **0.65 (0.44, 0.99)** |
| Social class in childhood |  |  |  |  |  |  |
| I/II | 991 (44.4) | 462 (37.2) | Reference | 506 (33.7) | 297 (29.9) | Reference |
| IIIa/b | 1112 (49.9) | 685 (55.2) | 1.19 (0.98, 1.45) | 869 (57.9) | 597 (60.1) | 1.05 (0.82, 1.36) |
| IV/V | 127 (5.7) | 95 (7.6) | 1.21 (0.78, 1.86) | 127 (8.5) | 99 (10.0) | 1.01 (0.63, 1.62) |
| Social class in adulthood |  |  |  |  |  |  |
| I/II | 1292 (60.5) | 693 (57.8) | Reference | 677 (46.3) | 479 (50.0) | Reference |
| IIIa/b | 697 (32.6) | 431 (35.9) | 1.04 (0.85, 1.28) | 625 (42.8) | 396 (41.3) | 0.92 (0.71, 1.17) |
| IV/V | 148 (6.9) | 76 (6.3) | 0.87 (0.57, 1.34) | 159 (10.9) | 83 (8.7) | 0.79 (0.49, 1.29) |
| Household size |  |  |  |  |  |  |
| <=3 persons | 209 (10.3) | 111 (10.4) | Reference | 129 (9.2) | 72 (8.5) | Reference |
| 4+ persons | 1813 (89.7) | 959 (89.6) | 1.02 (0.76, 1.37) | 1269 (90.8) | 780 (91.5) | 1.32 (0.86, 2.01) |
| Smoking during pregnancy |  |  |  |  |  |  |
| No | 1310 (59.0) | 697 (56.1) | Reference | 1022 (68.9) | 666 (68.0) | Reference |
| Any | 912 (41.0) | 545 (43.9) | 1.00 (0.82, 1.24) | 462 (31.1) | 313 (32.0) | 0.82 (0.62, 1.08) |
| Childhood smoke exposure |  |  |  |  |  |  |
| No | 762 (37.7) | 372 (34.8) | Reference | 355 (30.2) | 209 (28.3) | Reference |
| Any | 1259 (62.3) | 696 (65.2) | 0.99 (0.80, 1.23) | 821 (69.8) | 529 (71.7) | 1.08 (0.83, 1.41) |
| Adulthood smoking |  |  |  |  |  |  |
| No | 1234 (55.4) | 617 (49.5) | Reference | 864 (57.4) | 525 (52.7) | Reference |
| Any | 995 (44.6) | 629 (50.5) | **1.28 (1.06, 1.54)** | 640 (42.6) | 472 (47.3) | 1.00 (0.79, 1.27) |
| Other atopic history |  |  |  |  |  |  |
| Personal history of asthma | 714 (32.0) | 357 (28.7) | 0.83 (0.67, 1.02) | 541 (35.9) | 320 (32.1) | 0.82 (0.63, 1.05) |
| Personal history of allergic rhinitis/hay fever | 1242 (55.6) | 645 (51.8) | 0.99 (0.82, 1.20) | 752 (49.9) | 408 (40.9) | **0.71 (0.56, 0.90)** |
| Parental history of asthma or allergic rhinitis/hay fever | 715 (38.0) | 259 (26.7) | **0.58 (0.48, 0.72)** | ----- | ----- | ----- |
| Birth weight, mean (SD) | 3.3 (0.5) | 3.3 (0.5) | **0.82 (0.68, 0.98)*** | 3.3 (0.5) | 3.3 (0.5) | 1.07 (0.85, 1.34)* |
| Breastfeeding |  |  |  |  |  |  |
| No | 1153 (57.5) | 639 (60.1) | Reference | 380 (26.7) | 277 (31.6) | Reference |
| Any | 852 (42.5) | 425 (39.9) | 0.99 (0.82, 1.20) | 1045 (73.3) | 599 (68.4) | 0.87 (0.67, 1.13) |

Notes: *For every one kg increase in birth weight

**Supplemental Table 6.** Data on contact dermatitis, psoriasis, and physician-visits by cohort and age of atopic eczema (AE) onset

|  | 1970 Cohort | | | |  | 1958 Cohort | | | |
| --- | --- | --- | --- | --- | --- | --- | --- | --- | --- |
|  | No AE  N=8611 | Child-onset N=1972  (60% of those with AE) | Adult-onset N=1303  (40% of those with AE) | P-value* |  | No AE  N=10825 | Child-onset N=1313  (57% of those with AE) | Adult-onset N=1005  (43% of those with AE) | P-value |
| Contact dermatitis |  |  |  | <0.001 |  |  |  |  | <0.001 |
| No | 7891 (97.8) | 1755 (94.0) | 1153 (90.6) |  |  | 8755 (97.9) | 1056 (95.0) | 871 (91.6) |  |
| Any | 175 (2.2) | 112 (6.0) | 119 (9.4) |  |  | 188 (2.1) | 56 (5.0) | 80 (8.4) |  |
| Psoriasis |  |  |  | <0.001 |  |  |  |  | <0.001 |
| No | 7651 (97.0) | 1751 (95.1) | 1153 (92.5) |  |  | 10374 (96.3) | 1220 (93.2) | 925 (92.1) |  |
| Any | 233 (3.0) | 91 (4.9) | 94 (7.5) |  |  | 402 (3.7) | 89 (6.8) | 79 (7.9) |  |
| Reported seeing a physician in the past year** |  |  |  | 0.005 |  |  |  |  | 0.001 |
| No | N/A | 242 (61.3) | 287 (52.1) |  |  | N/A | 129 (65.8) | 308 (52.3) |  |
| Any | N/A | 153 (38.7) | 264 (47.9) |  |  | N/A | 67 (34.2) | 281 (47.7) |  |

Notes: *p-value for chi-square, **reported at age 30 in 1970 or age 42 in 1958

**Supplemental Table 7.** Sensitivity analysis results restricting to a subset of individuals with lower possibility of misclassification bias (i.e. reported seeing a MD in the last year and no history of contact dermatitis and psoriasis).

|  | Child-onset vs no AE  N= 13,099 | Adult-onset vs no AE  N=10,796 | Adult-onset vs child-onset AE  N=10,796 |
| --- | --- | --- | --- |
|  | Odds Ratio (95% CI) | | |
| Sex | | | |
| Male | Reference | Reference | Reference |
| Female | 1.06 (0.96, 1.17) | **2.13 (1.64, 2.76)** | **2.06 (1.57, 2.71)** |
| Ethnicity | | | |
| European, Caucasian | Reference | Reference | Reference |
| Other | 0.74 (0.52, 1.04) | 1.16 (0.53, 2.54) | 1.42 (0.61, 3.28) |
| Region of early childhood residence | | | |
| Southern England | Reference | Reference | Reference |
| Central England/Wales | 0.91 (0.81, 1.03) | 0.97 (0.64, 1.47) | 0.98 (0.63, 1.53) |
| N. England/Scotland | **0.75 (0.66, 0.84)** | 1.08 (0.68, 1.73) | 1.28 (0.77, 2.10) |
| Region of residence at age 42 | | | |
| Southern England | ----- | Reference | Reference |
| Central England/Wales | ----- | 1.26 (0.84, 1.89) | 1.26 (0.82, 1.94) |
| N. England/Scotland | ----- | 0.87 (0.55, 1.39) | 1.00 (0.61, 1.65) |
| Highest social class in childhood* | | | |
| I/II | Reference | Reference | Reference |
| III | **0.82 (0.74, 0.91)** | 0.94 (0.72, 1.23) | 1.11 (0.83, 1.47) |
| IV/V | **0.72 (0.58, 0.90)** | 0.99 (0.61, 1.61) | 1.32 (0.78, 2.24) |
| Highest social class in adulthood* | | | |
| I/II | ----- | Reference | Reference |
| III | ----- | 1.17 (0.90, 1.51) | 1.26 (0.95, 1.66) |
| IV/V | ----- | 1.15 (0.72, 1.81) | 1.45 (0.87, 2.39) |
| Household size in early childhood | | | |
| <=3 persons | Reference | Reference | Reference |
| 4+ persons | 1.04 (0.88, 1.23) | 1.02 (0.68, 1.53) | 0.99 (0.64, 1.54) |
| In utero smoke exposure | | | |
| No | Reference | Reference | Reference |
| Any | 0.92 (0.82, 1.03) | 1.01 (0.77, 1.33) | 1.10 (0.83, 1.48) |
| Childhood smoke exposure | | | |
| No | Reference | Reference | Reference |
| Any | 0.93 (0.83, 1.04) | 1.09 (0.82, 1.46) | 1.13 (0.83, 1.53) |
| Adulthood smoking | | | |
| No | ----- | Reference | Reference |
| Any | ----- | **1.58 (1.24, 2.01)** | **1.51 (1.16, 1.96)** |
| Other atopic history | | | |
| Asthma | **1.88 (1.69, 2.10)** | 1.21 (0.91, 1.60) | **0.67 (0.50, 0.90)** |
| Allergic rhinitis/hay fever | **1.79 (1.62, 1.98)** | **1.63 (1.27, 2.09)** | 0.92 (0.71, 1.21) |
| Birth weight | | | |
| Per kg increase | 1.08 (0.98, 1.19) | **0.70 (0.56, 0.89)** | **0.63 (0.49, 0.82)** |
| Breastfeeding | | | |
| No | Reference | Reference | Reference |
| Any | **1.13 (1.02, 1.26)** | 1.13 (0.87, 1.47) | 0.96 (0.73, 1.26) |

**Notes: ***Registrar General’s social class: I Professional, II Managerial and technical; III Skilled; IV Partly-skilled; V Unskilled.

**Supplemental Figure 1.** Eczema prevalence by calendar year

* Prevalence from age 0-5 for 1970 cohort and age 0-7 for 1958 cohort; bars represent 95% CIs.

**Supplemental Methods: British 1958 birth cohort biomedical examination and genotyping**

Total and specific IgE

At the age of 44-45 years, the 1958 cohort were followed up with a biomedical examination and blood sampling (E1), from which a DNA collection was established as a nationally representative reference panel. In blood samples collected at this adult follow up, the total concentration of serum IgE antibodies and the presence of specific IgE to house dust mite, mixed grass pollen and cat fur were ascertained by Hytec enzyme immunoassay, with a detection threshold of 0.35 kU/L. (E2)

Filaggrin null mutations

The four common null mutations of the filaggrin (*FLG*) gene that have been associated with risk of atopic dermatitis (E3, E4) were genotyped directly by LGC Genomics using KASP^TM^ genotyping technology. Filaggrin null status was defined as the presence of one or more risk variants of rs61816761 (R501X), rs150597413 (S3247X), rs558269137 (2282del4) or rs138726443 (formerly rs386430951).

Genome-wide typing, imputation and generation of non-FLG genetic risk score

Three non-overlapping subsets of the DNA collection from cohort members of white European ethnicity were genotyped by the Wellcome Trust Case-Control Consortium (WTCCC) (E5); the Type 1 Diabetes Genetics Consortium (T1DGC) (E6); and the GABRIEL consortium (E7). Genotyping was performed using the Illumina 550K array (WTCCC1 and T1DGC), the Illumina 610K array (GABRIEL) or the Illumina 1M array (WTCCC2). A set of SNPs common to these arrays were used for imputation against the March 2012 (phase 1, version 3) release of the 1000-genomes reference haplotypes for all ancestries. Pre-imputation phasing was performed using MACH v1.0.18 and imputation was performed using Minimac (version dated 16 November 2012).

The following 29 variants outside the FLG region were selected for inclusion in a polygenic risk score, based on previously published associations with atopic dermatitis:

rs7927894_T (risk-associated variant T) (C11orf30 / 11q13.5) (E8);

rs6010620_G (TNFRSF6B / 20q13.33) and rs7701890_G (TMEM232 / 5q22.1) (E9);

rs479844_G (OVOL1 / 11q13.1), rs2164983_A (ACTL9 / 19p13.2) and rs2897442_C (KIF3A / 5q31) (E10);

rs13015714_G (IL1RL1-IL18RAP / 2q12), rs114764276_A (GPSM3 / 6p21.3), rs878860_C (OR10A3-NLRP10 / 11p15.4), rs6780220_C (GLB1 / 3p21.33), rs12634229_C (CCDC80 / 3q13.2), rs4722404_C (CARD11 / 7p22), rs10995251_C (ZNF365 / 10q21.2) and rs16999165_A (CYP24A1-PFDN4 / 20q13) (E11);

rs17389644_A (IL2-IL21 / 4q27), rs12295535_T (PRR5L / 11p13), rs2041733_T (CLEC16A-DEXI / 16p13.13) and rs16948048_G (ZNF652 / 17q21.32) (E12);

rs12153855_T (TNXB / 6p21) (E13);

rs7127307_T (ETS1 / 11q24.3), rs2227483_T (IL22 / 12q15); rs2143950_T (PPP2R3C / 14q13.2), rs7146581_C (TRAF3/14q32.32), rs17881320_T (STAT3 / 17q21.2), rs11657987_T (SOCS3 /17q25.3), rs112111458_A (CD207 / 2p13.3), rs1057258_C (INPP5D / 2q37.1), rs10214237_T (IL7R / 5p13.2) and rs6473227_C (ZBTB10 / 8q21.13) (E14).

A non-FLG genetic risk score was generated as the sum of imputed allele dosages for the risk-associated variant at each of these SNPs.

References

E1. Strachan DP, Rudnicka AR, Power C, Shepherd P, Fuller E, Davis A, et al. Lifecourse influences on health among British adults: effects of region of residence in childhood and adulthood. Int J Epidemiol. 2007;36(3):522-31.

E2. Butland BK, Strachan DP. Asthma onset and relapse in adult life: the British 1958 birth cohort study. Ann Allergy Asthma Immunol. 2007;98(4):337-43.

E3. Palmer CN, Irvine AD, Terron-Kwiatkowski A, Zhao Y, Liao H, Lee SP, et al. Common loss-of-function variants of the epidermal barrier protein filaggrin are a major predisposing factor for atopic dermatitis. Nat Genet. 2006;38(4):441-6.

E4. Irvine AD, McLean WH, Leung DY. Filaggrin mutations associated with skin and allergic diseases. N Engl J Med. 2011;365(14):1315-27.

E5. Consortium WTCC. Genome-wide association study of 14,000 cases of seven common diseases and 3,000 shared controls. Nature. 2007;447(7145):661-78.

E6. Barrett JC, Clayton DG, Concannon P, Akolkar B, Cooper JD, Erlich HA, et al. Genome-wide association study and meta-analysis find that over 40 loci affect risk of type 1 diabetes. Nat Genet. 2009;41(6):703-7.

E7. Moffatt MF, Gut IG, Demenais F, Strachan DP, Bouzigon E, Heath S, et al. A large-scale, consortium-based genomewide association study of asthma. N Engl J Med. 2010;363(13):1211-21.

E8. Esparza-Gordillo J, Weidinger S, Folster-Holst R, Bauerfeind A, Ruschendorf F, Patone G, et al. A common variant on chromosome 11q13 is associated with atopic dermatitis. Nat Genet. 2009;41(5):596-601.

E9. Sun LD, Xiao FL, Li Y, Zhou WM, Tang HY, Tang XF, et al. Genome-wide association study identifies two new susceptibility loci for atopic dermatitis in the Chinese Han population. Nat Genet. 2011;43(7):690-4.

E10. Paternoster L, Standl M, Chen CM, Ramasamy A, Bonnelykke K, Duijts L, et al. Meta-analysis of genome-wide association studies identifies three new risk loci for atopic dermatitis. Nat Genet. 2011;44(2):187-92.

E11. Hirota T, Takahashi A, Kubo M, Tsunoda T, Tomita K, Sakashita M, et al. Genome-wide association study identifies eight new susceptibility loci for atopic dermatitis in the Japanese population. Nat Genet. 2012;44(11):1222-6.

E12. Ellinghaus D, Baurecht H, Esparza-Gordillo J, Rodriguez E, Matanovic A, Marenholz I, et al. High-density genotyping study identifies four new susceptibility loci for atopic dermatitis. Nat Genet. 2013;45(7):808-12.

E13. Weidinger S, Willis-Owen SA, Kamatani Y, Baurecht H, Morar N, Liang L, et al. A genome-wide association study of atopic dermatitis identifies loci with overlapping effects on asthma and psoriasis. Hum Mol Genet. 2013;22(23):4841-56.

E14. Paternoster L, Standl M, Waage J, Baurecht H, Hotze M, Strachan DP, et al. Multi-ancestry genome-wide association study of 21,000 cases and 95,000 controls identifies new risk loci for atopic dermatitis. Nat Genet. 2015;47(12):1449-56.
